# Diffuse lipofibromatosis with hand bone involvement: expanding the clinical spectrum: A case report and literature review

**DOI:** 10.1016/j.jpra.2026.01.038

**Published:** 2026-01-29

**Authors:** Povilas Jurgutavičius, Mindaugas Minderis, Mykolas Udrys, Giedrė Stundžaitė-Baršauskienė

**Affiliations:** a Faculty of Medicine, Vilnius University, Vilnius, Lithuania; b Center of Plastic and Reconstructive Surgery, Vilnius University Hospital Santaros Klinikos, Vilnius, Lithuania; c Department of Physiology, Biochemistry, Microbiology, and Laboratory Medicine, Institute of Biomedical Sciences, Vilnius University, Vilnius, Lithuania; d Clinic of Rheumatology Orthopedics-Traumatology and Reconstructive Surgery, Institute of Clinical Medicine Faculty of Medicine Vilnius University, Vilnius, Lithuania

**Keywords:** Diffuse lipofibromatosis, Bone involvement, Upper limb deformity, Hand reconstruction

## Abstract

**Introduction:**

Lipofibromatosis (LF) is a rare, benign fibroblastic–adipocytic tumor of childhood, classified by the World Health Organization as an intermediate (locally aggressive) soft tissue neoplasm. Due to its rarity, diagnostic criteria and management guidelines remain poorly defined. This article presents an exceptionally rare case of diffuse upper limb LF with osseous involvement in adulthood and provides a comprehensive literature review to contextualize current knowledge on its epidemiology, etiology, clinical presentation, diagnostics, and treatment.

**Case presentation:**

A 48-year-old woman presented with progressive deformity of the right hand following lifelong, slowly enlarging soft-tissue masses. Imaging revealed extensive fusion of the distal radius, wrist, and thumb. Histological examination confirmed the diagnosis of LF. Osseous involvement of LF is exceedingly uncommon, and this case represents one of the first documented instances of extensive skeletal involvement. A two-stage reconstructive approach—tumor resection followed by a subsequent corrective thumb osteotomy—led to substantial recovery of hand function, as reflected in a 15,9-point improvement in the disabilities of the arm, shoulder and hand score.

**Conclusions:**

This article offers valuable insights into diagnostics and potential reconstructive approaches for similar cases. The accompanying literature review further broadens the understanding of this rare clinical entity.

## Introduction

Lipofibromatosis (LF) is a rare, benign soft tissue tumor typically present in early childhood.^1^ Historically, LF was considered a variant of fibromatosis or fibrous hamartoma of infancy. However, in 2000, Fetsch et al. proposed its classification as a distinct clinicopathologic entity.^2^ This distinction was recognized in the 2002 World Health Organization classification of soft tissue tumors,^3^ which was later reclassified as an intermediate (locally aggressive) fibroblastic/myofibroblastic tumor.^4^ Because LF is a rare pathology, no standardized diagnostic or treatment guidelines currently exist. Consequently, unusual cases often raise important clinical questions. This article aims to present an extraordinarily rare case of diffuse LF of the hand, distinguished by adult presentation, severe osseous involvement localized to the hand, and resulting functional impairments. Such a constellation of features has not been well documented in the literature and expands the current understanding of the skeletal manifestations, long-term tumor behavior, and possible treatment options for LF. In addition, a focused review of the literature is provided, summarizing the epidemiology, etiology, clinical presentation, diagnostic approaches, and management of this uncommon entity.

## Literature review

### Epidemiology

Approximately 18% of LF cases are congenital,^1^ the remainder are typically diagnosed between 11 days and 12 years of age.^2^ Rare cases have been reported during adolescence.^5^ While some studies suggest a male predominance with a male to female ratio of approximately 2,7:1,^1^ other reports indicate no significant gender predilection.^2^ The most common tumor locations include hands, arms, legs, and feet,^1^^,^^2^ with less frequent involvement of the chest, abdomen, back, and head and neck.^2^ Rare orbital^1^^,^^2^^,^^6^ as well as scalp,^7^ central nervous system (CNS),^8^ and groin/labia^9^ involvements have also been described.

### Etiology

Although the exact etiology of this rare condition remains unknown, it is believed that both adipose tissue and spindle cell components contribute to the neoplastic process, with spindle cells appearing to represent the main proliferative element.^2^ Previous studies have implicated dysregulation of the PI3K–AKT–mTOR pathway,^10^ and a chromosomal translocation involving the fourth, sixth, and ninth chromosomes has been reported.^11^ Additionally, LF of the lower limb has been described in association with multiple congenital anomalies, including syndactyly, bilateral complete cleft lip and palate, trigonocephaly, and an atrial septal defect.^12^

### Clinical presentation

Tumors typically present as slow-growing, asymptomatic, or mildly painful masses ranging from 1 to 7 cm in diameter. They are characteristically firm, rubbery in consistency. In rare instances may exhibit rapid growth.^2^ Several authors have noted accelerated tumor growth in the second decade of life.^13^ Exceptional cases have been documented with tumors reaching sizes up to 35 cm^14^ or diffusely involving the entire limb.^15^, ^16^, ^17^ Due to its tendency to grow, tumors can be locally destructive. They may infiltrate or entrap adjacent structures, including blood vessels, nerves, skin adnexa, and skeletal muscles, causing tenderness, pain, contractures, and restricted movement.^2^ Increased hair growth overlying the lesion was noticed in one case,^18^ and skin ulceration over the lesion was reported in another case.^19^ In rare instances, progressive enlargement leads to bone reshaping: bowing of the tibia and fibula has been documented,^13^^,^^17^ as well as remodeling of hand bones and syndactyly in cases of digital nerve territory–oriented LF.^13^^,^^14^ Severe deformity of all right upper limb bones due to chronic pressure has been described, including narrowing and tapering of the distal humerus and bowing and thinning of the radius and ulna.^16^ Although significant functional impairment is rare, orbital LF has been associated with impaired visual acuity and restricted ocular motility (20), LF involving the CNS has been associated with loss of spontaneous respiration and quadriparesis.^8^

### Diagnostics

The tumor’s broad and often nonspecific clinical presentation, which may resemble numerous other soft-tissue pathologies, underscores the importance of instrumental diagnostic techniques. Radiologic evaluation has limited diagnostic specificity due to overlap with other fibrofatty lesions.^21^ A nonspecific well-circumscribed hyperechoic mass is typically seen in ultrasound examination.^6^ LF in plain radiographs is unremarkable or may reveal as a radiolucent lesion without calcifications. Magnetic resonance imaging (MRI) generally demonstrates a predominantly fatty, lobulated lesion with interspersed low-signal fibrous septa and variable contrast enhancement.^1^ In some cases, computed tomography (CT) or MRI may demonstrate erosive changes of adjacent bones.^16^^,^^20^ Ultimately, a definitive diagnosis requires histopathological examination. Fine needle aspiration cytology has been utilized as a minimally invasive diagnostic tool in at least one reported case, where the presence of skeletal muscle fibers admixed with benign fibroblastic and adipocytic elements was considered suggestive of LF.^22^ Currently, excisional biopsy remains the most used and reliable diagnostic method. Grossly, LF appears yellow, tan, or white, with a fatty or fibrofatty appearance and irregular margins, often described as lobulated,^2^ in some cases limited by a thin fibrous capsule,^9^^,^^11^ in others, infiltrated in subcutaneous tissues, muscles^20^^,^^23^ or bones. A minority of cases have been reported as highly vascular.^7^^,^^23^ Histologically, LF is characterized by abundant mature adipose tissue (usually >50% of the lesion) interspersed with fibroblastic spindle cells and collagenous septa.^2^ Several investigators employed immunohistochemical analysis to identify the expression of CD34, CD99, SMA, and BCL-2, which demonstrated positivity in cases of LF.^2^ However, subsequent research has suggested that these markers lack specificity and offer limited diagnostic value.^24^

### Management

Although benign, LF may behave in a locally destructive manner and cause significant cosmetic or functional impairment, requiring clinical intervention. Non-surgical management of LF remains limited. Chemotherapy of five doxorubicin and ifosfamide cycles has been attempted without success,^7^ and radiotherapy has similarly shown no proven benefit.^2^ Consequently, the treatment of choice is complete surgical excision, as the residual tumor demonstrates a high tendency for local recurrence. It is known from Fetsch et al. study that 72% of resected tumors recur. Recurrence or persistent disease appears to be more common in patients with congenital onset, male sex, tumors located on the hands or feet, incomplete excision, and mitotic activity within the fibroblastic component.^2^

Extensive, infiltrative LF involving surrounding anatomical structures often limits the ability to perform a radical excision. Due to the infiltrative nature of the tumor, negative-margin excision is achievable in only 78,3% of cases.^2^ Incomplete excision of LF is most frequent in limbs,^1^^,^^2^ as wide excision of extensive lesions carries a risk of compromising limb viability and functionality.^16^ Therefore, in the setting of extensive limb involvement, alternative management approaches are necessary, including careful observation, debulking, or, in rare and severe cases, amputation.

Given the characteristically slow growth of LF, the lesion rarely causes severe morbidity. Accordingly, in cases of diffuse limb involvement, a watchful waiting approach may be appropriate unless the tumor leads to functional impairment.^15^ Several cases have documented long-term stability of incompletely resected lesions, supporting tumor debulking^16^^,^^20^ in cases of tumors that significantly impair function or have a high potential to do so if allowed to grow.^18^ The risks of surgical complications and tumor-related morbidity must be carefully weighed, as unsuccessful or overly aggressive interventions can result in limb loss. Amputation for LF is rare and is typically a last resort if the tumor is causing significant functional impairment of the limb, which cannot be improved with limb-sparing surgery.^14^

## Case presentation

A 48-year-old woman presented with progressive deformities of the right hand. The hand was normal at birth. During the first year of life, multiple soft tissue tumors began to appear in the upper limb, including the shoulder, arm, forearm, and thumb. The lesions developed in a chain-like pattern and gradually spread over time, remaining confined to the upper limb. The thumb became increasingly deformed and eventually ankylosed. There was no family history of similar deformities. The patient underwent surgical excisions at ages 3, 8, and 21 years. At ages 3 and 8, tumors in the upper arm were resected, while at age 21, a tumor between the first and second metacarpals was removed. Histological analysis after each procedure confirmed the diagnosis of LF. Although the patient adapted to the deformities, her condition worsened in recent years due to progressive enlargement of the thumb, which increasingly interfered with daily activities such as working, eating, and writing. The patient evaluated her functional disability using the Disabilities of the Arm, Shoulder and Hand (DASH) questionnaire, scoring 44,2 points out of 100.

**Figure 1:**
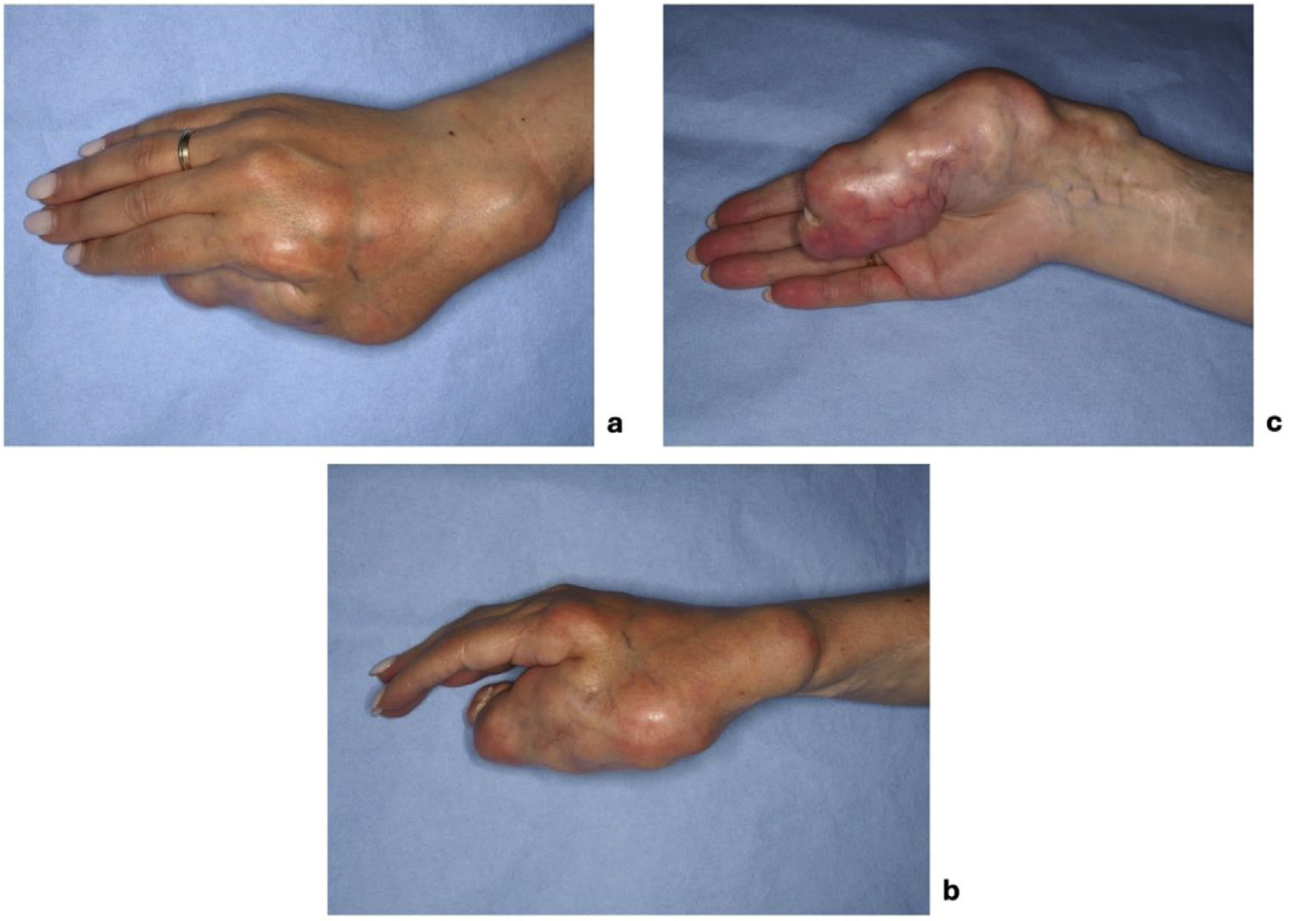
The patient's hand photographs showing tumorous masses before surgical treatment (a) – on the back radial side of the hand, (b) – on the dorsal side of the thumb and forearm, (c) – on the volar side of the thumb.

**Figure 2:**
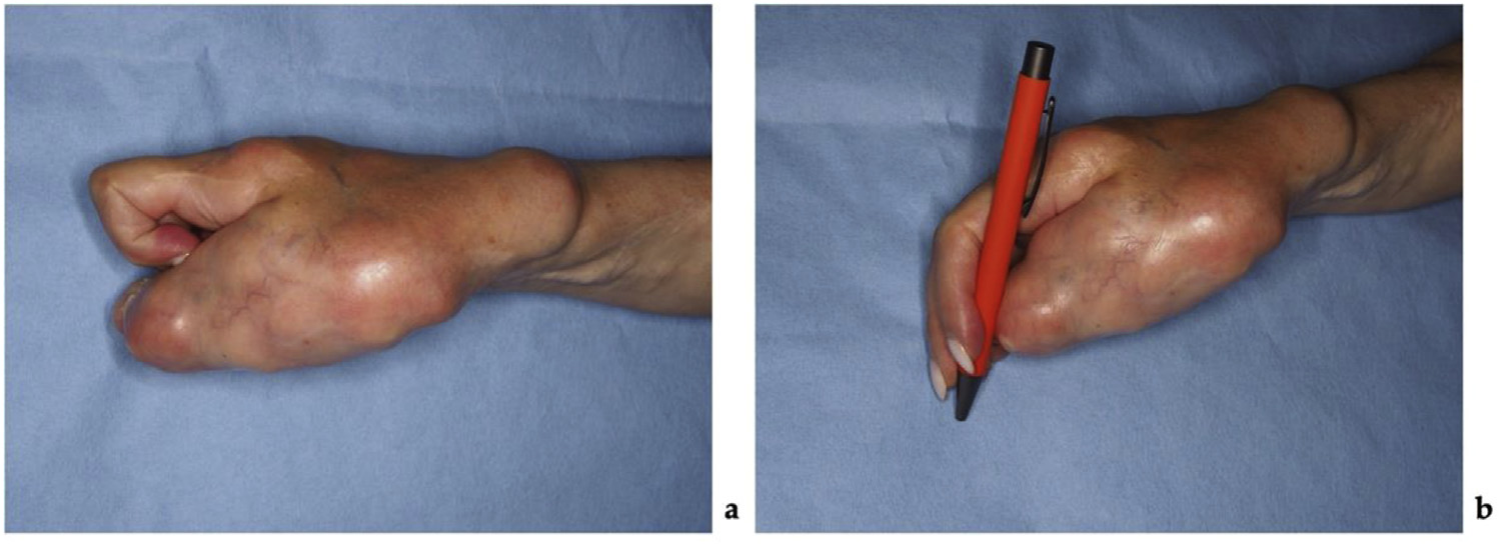
The patient's hand photographs show tumorous masses (a) overlapping fingers when trying to squeeze a fist and (b) interfering with writing.

**Figure 3:**
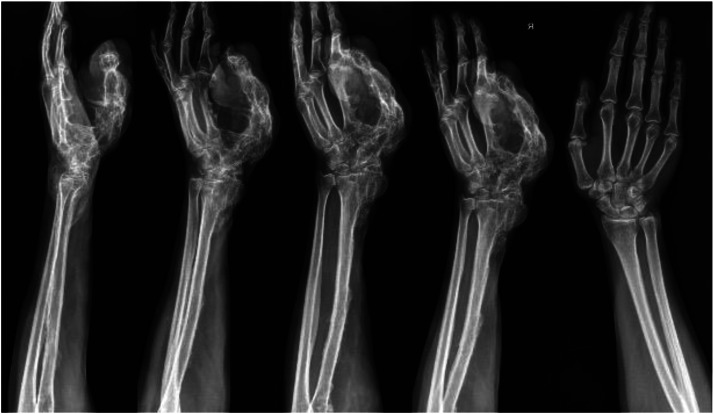
The patient's hand radiographs before surgical treatment.

On clinical examination, multiple connected soft tissue tumors were observed in the right upper limb. Soft tissue masses enveloped the thumb and spread to the radial side of the forearm with poorly defined boundaries. Hard, bony-like masses were protruding over the distal end of the radius (Figure 1). Masses limited thumb and wrist function. Wrist ankylosis was noted, with less than 5° of motion remaining in both sagittal and frontal planes. The thumb was severely affected, with complete loss of movement. It was positioned at an unfavorable 60° angle at the MCP joint, lying centrally in the palm without adherence, and preventing flexion of the other fingers during fist formation (Figure 2). The shoulder, elbow, and joints of the second to fifth fingers were unaffected. Sensation was not intact in any finger or hand. Only the tip of the affected thumb, where the tumor mass elevated the nail, was more sensitive to palpation. No other neurovascular deficits were noticed. Radiographic imaging revealed involved bones. Deformities were evident on the radial side of the hand, with fusion of the distal radius, wrist, and thumb, further distinguishing this case from the typical presentation of soft tissue tumor (Figure 3).

A staged surgical approach was planned, with the primary objective of improving functional hand use by optimizing thumb position and widening the first web space. The first stage focused on excision of the tumor surrounding the thumb, repositioning the thumb into a more functional alignment, and creating adequate first web space, which is essential for effective grasp and object manipulation. This step was intended to establish a functional foundation for hand use and to facilitate subsequent surgical procedures. Following recovery from this procedure, a second stage was scheduled to address the soft tissue masses on the volar aspect and the bony prominences on the dorsal side of the hand.

**Figure 4:**
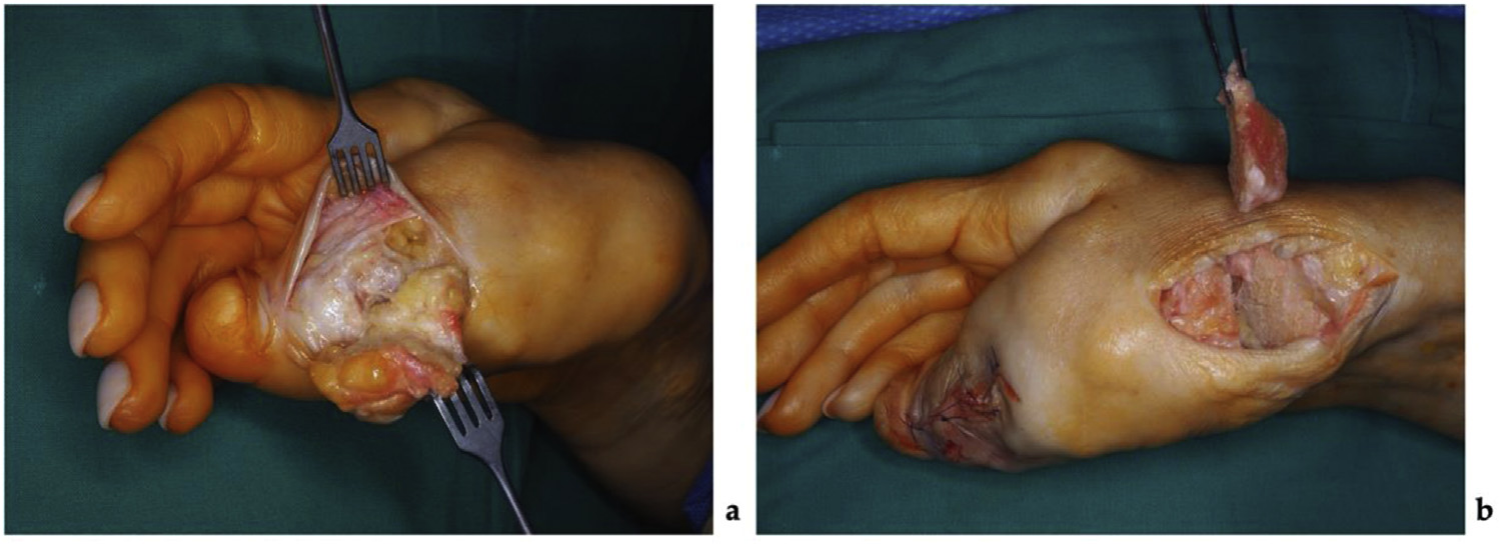
Photographs showing the first stage of the surgical management: (a) – a tumorous mass of the affected thumb resection, (b) – resection of the first metacarpal bone.

Under regional anesthesia, the patient underwent partial tumor resection of the right hand and a thumb osteotomy with fixation. A 3,5 × 4 cm fat-like tumor with a clear boundary from the skin, but indistinct lateral margins, and tightly fused with the bone, was excised from the tip of the right thumb through a zigzag-type incision. An additional 4 × 5 × 1 cm mass was excised through an incision at the thumb at the level of the carpometacarpal joint. Visualized thumb carpometacarpal joint was severely affected and no longer functional. A triangular resection of the first metacarpal bone was performed to create a 40° defect. The thumb was then repositioned and fixated using four Kirschner wires (Figure 4). The resected soft tissue and bone fragments were re-evaluated histologically. The fragments comprised mature adipose tissue with fibrous septa of varying widths, containing bundles of monomorphic spindle-shaped fibroblasts. The histological appearance was most consistent with soft tissue and bone LF. The total operative time was 2 h and 15 min. A passive drain was placed to prevent hematoma formation, and the thumb was immobilized in a custom-molded plastic splint for 4 weeks to maintain stability and protect the reconstruction.

**Figure 5:**
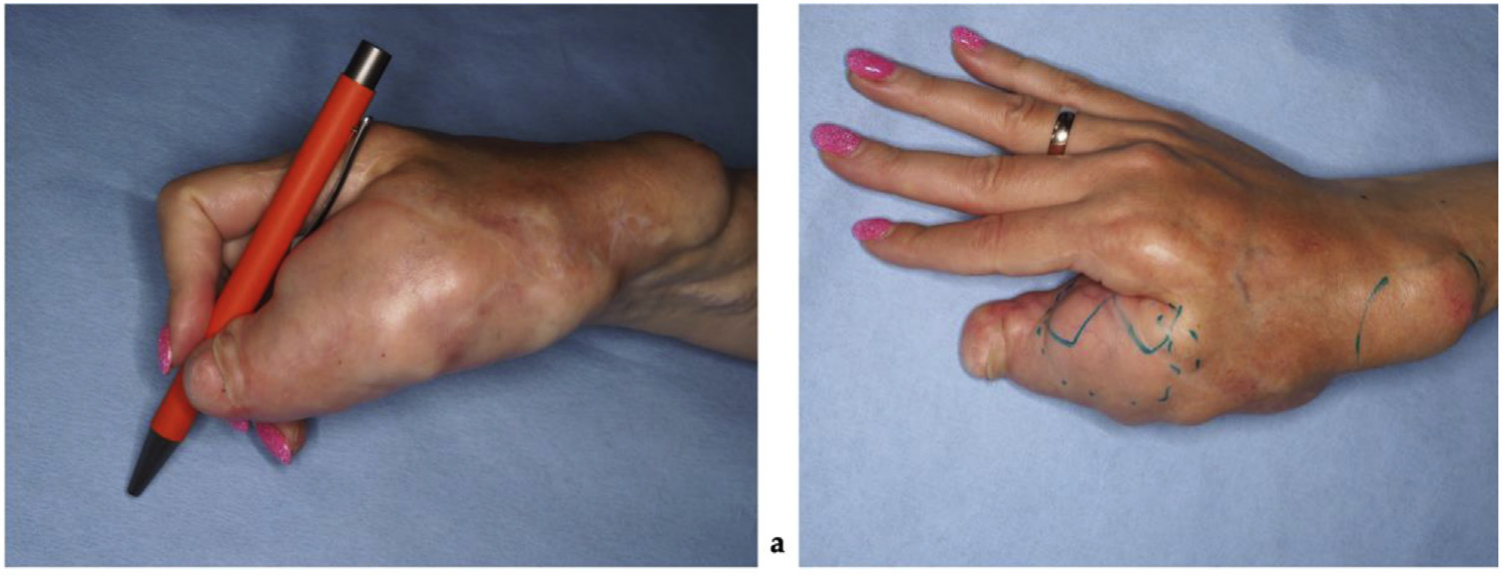
Photographs taken 2 months after the first stage of surgical management showing: (a) the patient's ability to hold a pen improved, (b) decreased masses over the thumb and between the first and second fingers.

**Figure 6:**
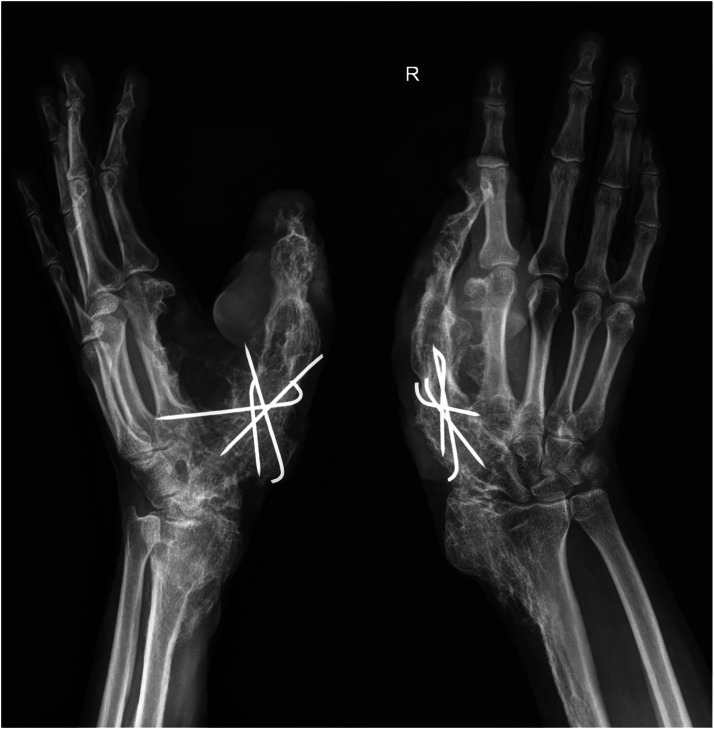
X-ray pictures of the patient’s right hand 2 months after the first surgical stage, before the K-wires removal.

At the 2 months of follow-up, significant improvement was observed, with no tumor progression or postoperative complications. There was increased space between the first and second fingers and evidence of bone healing following osteosynthesis (Figure 5,6).

**Figure 7:**
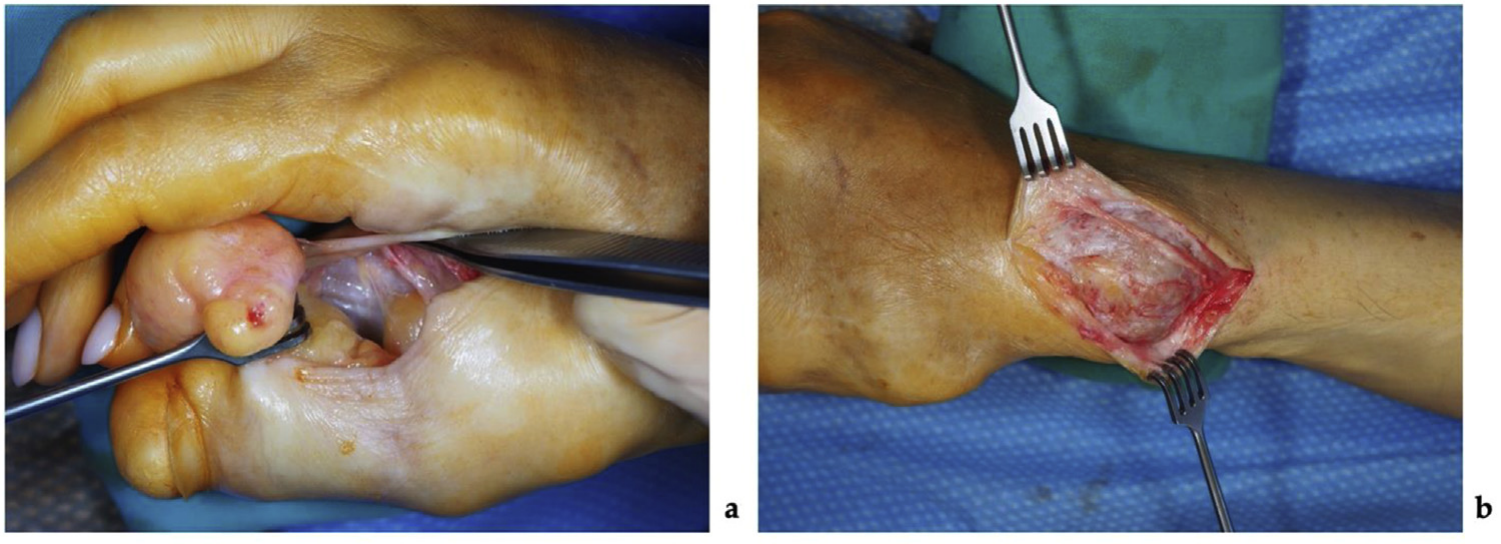
Photographs showing the second stage of the surgical management: (a) – tumor resected from the first intermetacarpal space, in the bottom, the neurovascular bundle can be seen; (b) – the sensory radial nerve branch was found tightly fused with the osseous structure of the tumor and carefully separated.

After 8 months, the patient underwent a second surgery under regional anesthesia. Incisions were made over the thumb's dorsolateral aspect and the radius's distal end. Through the dorsolateral incision, both dorsal and volar thumb tumors were excised. Lesions were arranged in a chain-like pattern, appeared well demarcated externally, but were firmly adherent to the underlying bone. The neurovascular bundle was displaced volarly toward the skin by the masses. At the distal radius, a separate incision revealed a nerve fused to the bony tumor mass; the nerve was carefully separated from the osseous tissue with a scalpel (Figure 7). The bony mass was then excised using an oscillating saw, and irregularities of the resected bone were smoothed. Neurovascular structures were intact at the end of the procedure, with no evidence of vascular or neural deficit. The excised bone and soft tissue specimens were submitted for histologic examination, which again confirmed LF. The operation lasted 1 h and 40 min, and both bone wax and active drainage were used to control bleeding and prevent hematoma formation.

**Figure 8:**
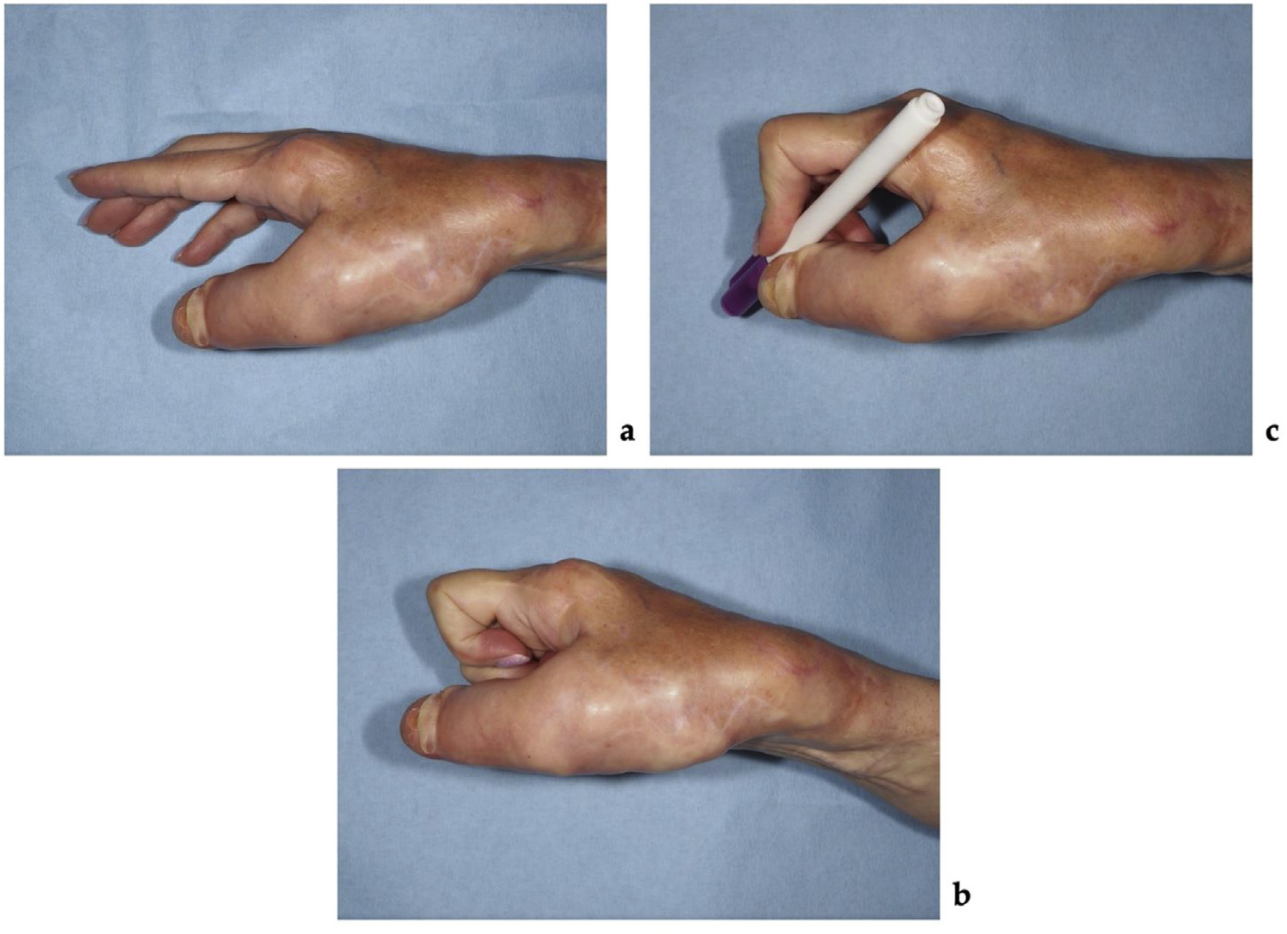
Two months after the final surgical stage: (a) widened first–second interdigital space; (b) patient attempting to clench a fist; (c) patient holding a pencil.

Two months after the second stage surgery, the patient had fully healed without any complications (Figure 8). She completed the DASH questionnaire again, showing an improvement by 15,9 points. The patient rated her satisfaction with the outcome as 10 out of 10. Functional improvement was attributed to a combination of repositioning of the thumb into a more functional alignment, resulting in effective widening of the first web space, and tumorous masses debulking. Despite persistent thumb immobility, the widened first web space enabled more effective hand use. The patient reported more frequent use of the affected right hand in daily activities compared with the preoperative period. She was able to hold a pen and write, flex the second to fifth fingers into a fist without limitation, and manipulate smaller objects that she had been unable to handle preoperatively. Additionally, she reported marked improvement in fine functional tasks such as writing, object handling, and wearing gloves or long, tight-sleeved clothing.

## Discussion

Several soft tissue and lymphatic lesions, including lipomatosis, infiltrative liposarcoma, lipoblastomatosis, fibrous hamartoma of infancy, desmoid-type fibromatosis, and lymphatic malformation, may mimic diffuse LF. Unlike the present case, lipomas usually occur in adults, are uncommon in the hand, and rarely recur after excision.^25^ The slow, indolent course beginning in infancy makes liposarcoma unlikely.^26^ Although fibrous hamartoma of infancy shares some features with LF, it typically arises within the first year of life and, similar to desmoid-type fibromatosis, is rarely seen in the hand.^27^^,^^28^

Clinically, soft tissue tumors are often difficult to distinguish from one another, necessitating the use of additional diagnostic tools. MRI is the most appropriate modality for evaluating soft tissue lesions; however, it lacks specificity in differentiating between various tumor types, as many contain adipose components that appear hyperintense on both T1- and T2-weighted sequences. Moreover, both clinical and imaging findings are often nonspecific, particularly in atypical or mixed-pattern lesions. Therefore, a definitive diagnosis relies on histopathological examination, which remains the gold standard.

Diffuse upper extremity involvement may be confused with more common causes of a swollen limb, such as lymphatic malformation. Similar to LF, lymphatic malformation primarily occurs in children and may recur after excision.^29^^,^^30^ Differently, it presents in lymphatic-rich areas, while involvement of the upper extremity and particularly the hand is rare.^30^ They are often distinguished by a bluish discoloration of the overlying skin, the presence of vesicles, and a tendency toward recurrent skin infections, features not observed in LF.^29^ On MRI or ultrasound, lymphatic malformations typically demonstrate multiple cystic cavities filled with fluid, and they exhibit characteristic immunohistochemical and histological features provided in Table 1.^31^

**Table 1:** Differential diagnosis of soft tissue tumors clinically similar to diffuse lipofibromatosis.

Tumor or anomaly	Age of incidence	Anatomic sites	Biological behavior	MRI features	Histological findings
Lipoma	50–60^25^	Neck, trunk, proximal extremities^25^	Benign^25^	Thin fibrous capsule, fibrous septa, and fat lobules^25^	Mature adipose tissue separated by fibrous septa and blood vessels^25^
Liposarcoma	60–70^32^^,^^33^	Retroperitoneum, proximal extremities^32^^,^^33^	Malignant^32^^,^^33^	Thick fibrous septa, nodules, or areas of necrosis^32^	Atypical adipose tissue and/or lipoblasts, non-lipogenic sarcomatous areas^32^^,^^33^
Lipoblastoma	< 5^34^	Extremities, trunk^35^	Benign^34^^,^^35^	Multilobulated fatty mass with some linear septations^34^	Lobulated lesions containing lipoblasts, plexiform capillary network, and myxoid stroma^35^^,^^36^
Fibrous hamartoma of infancy	< 2^27^	Axilla, trunk, upper arm, external genitalia^27^^,^^37^	Benign^27^^,^^37^	Organized arrangement of fat with interspersed, heterogeneous soft tissue bands^27^	Triphasic morphologic appearance of fibrous trabeculae, mature adipose cells, and immature mesenchymal tissues^37^
Desmoid-type fibromatosis	15–60^38^	Abdominal wall, intra-abdominal cavity, limbs^38^^,-^^40^	Locally aggressive^38^	The lesions show homogenous isointensity, similar to skeletal muscle, on T1-weighted sequences, and heterogenous iso to hyperintensity on T2-weighted sequences, depending on the proportion of collagen fibers, spindle cells, myxoid components, and extracellular matrix^40^	Broad, sweeping fascicles of uniform, fibroblastic cells with a variable amount of collagenous stroma^39^
Lymphatic malformation	< 2^30^	Head, axilla, neck^29^^,^^30^	Recure after excision^29^^,^^30^	Not enhancing with contrast, T1 weighted -low intensity, T2 weighted -high intensity^31^	Thin-walled channels lined by flat endothelium, devoid of muscle, containing proteinaceous material,^31^ positive immunohistochemistry^29^

After careful consideration of the differential diagnoses, the clinical and histopathological findings were most consistent with LF. The present case further highlights previously unreported aspects of osseous involvement in LF and demonstrates that successful surgical management of affected bones is possible. To date, only one case has described correction of a tibial deformity associated with LF in a 2-year-old child, in which the bone morphology differed substantially from that observed in our patient.^17^ Our findings, therefore, represent one of the first descriptions of severe osseous involvement in an adult and provide novel insight into potential treatment strategies. Intraoperatively soft tissue tumor was found firmly adherent to the underlying bone, which complicated its separation from normal osseous tissue. Examination of the affected bones showed marked surface irregularities, including multiple prominences, sharp ridges, and shallow depressions. The cortical layer was largely missing, and the underlying bone was unusually soft, allowing it to be incised with a scalpel. Corrective thumb osteotomy with four K-wires achieved stable and well-aligned fixation. Radiographs at 2 months demonstrated progressive consolidation, allowing K-wire removal. By 5 months, complete osseous union had been achieved with the absence of comorbidities. Taken together, these findings suggest that even in cases of extensive bone involvement, reconstruction can be successful, and the experience gained from this case may serve as a valuable reference for future attempts to manage similar presentations.

Predicting the impact of LF on the neurovascular structures of the fingers remains challenging. In the presented case, no clinical signs of vascular or neural compromise were observed; however, Fetsch et al., in a series of 45 patients, reported infiltration or entrapment of nerves and/or vessels in nearly every case.^2^ Therefore, lack of overt clinical signs should be interpreted cautiously. Intraoperatively, considerable attention was devoted to identifying and preserving the neurovascular bundles. During the second stage of surgery, these structures were found to be markedly displaced, compressed against the overlying skin, or fused with the tumor itself, illustrating the degree of anatomical distortion that can occur. These findings emphasize the need for meticulous surgical planning and dissection in similar cases, as neurovascular involvement should be anticipated even when not clinically apparent.

As LF is characterized by enlargement and recurrence, long-term follow-up is essential for appropriate management. Previous studies have reported variable time intervals until recurrence, with Fetsch et al. describing an average of 3 years^2^ and Boos et al. noting recurrences at 7 and 15 months after resection.^1^ Some cases indicate that no further recurrence was observed after resection of previously recurred tumors for 5 to 10 years.^2^ The case reported in this article is distinctive in demonstrating slow but continuous tumor progression over 4 decades, with only limited recurrences despite multiple resections. Resections performed at ages 3 and 8 in the upper arm and thumb regions resulted in no recurrence or further enlargement at the operated sites. However, the hand lesions continued to grow slowly and steadily until age 21, when the interdigital mass was excised. The thumb-associated tumors persisted with slow progression, though the patient reported a marked acceleration in hand mass enlargement during the past decade. Throughout treatment and follow-up after surgeries, no rapid tumor growth or recurrence was observed. This clinical course suggests that LF may follow a prolonged and indolent trajectory, although periods of more rapid progression can occur. Given the known tendency of LF toward slow progression and recurrence, long-term follow-up is essential, even after apparently successful surgical treatment. Surveillance should include regular clinical examinations, and patients should be counseled regarding the risk of late recurrence and its potential functional impact. In addition, patients should be educated to recognize signs of disease progression and offered individualized management strategies tailored to tumor behavior, anatomical involvement, and symptom burden.

## Informed consent

Written informed consent was obtained from the patient for their anonymized information to be published in this article.

## IRB approval

Local IRB considers case reports do not require IRB review.

## Funding

The authors received no financial support for the research, authorship, and/or publication of this article.

## Declaration of competing interest

The authors declared no potential conflicts of interest with respect to the research, authorship, and/or publication of this article.
